# Identification of genetic variants associated with dengue or West Nile virus disease: a systematic review and meta-analysis

**DOI:** 10.1186/s12879-018-3186-6

**Published:** 2018-06-22

**Authors:** Megan E. Cahill, Samantha Conley, Andrew T. DeWan, Ruth R. Montgomery

**Affiliations:** 1 Yale University School of Public Health, New Haven, CT USA; 2 Yale University School of Nursing, West Haven, CT USA; 3 Yale University School of Medicine, New Haven, CT USA

**Keywords:** Dengue virus, West Nile virus, Disease severity, Genetic variation, Meta-analysis, Single nucleotide polymorphism

## Abstract

**Background:**

Dengue and West Nile viruses are highly cross-reactive and have numerous parallels in geography, potential vector host (*Aedes* family of mosquitoes), and initial symptoms of infection. While the vast majority (> 80%) of both dengue and West Nile virus infections result in asymptomatic infections, a minority of individuals experience symptomatic infection and an even smaller proportion develop severe disease. The mechanisms by which these infections lead to severe disease in a subset of infected individuals is incompletely understood, but individual host differences including genetic factors and immune responses have been proposed. We sought to identify genetic risk factors that are associated with more severe disease outcomes for both viruses in order to shed light on possible shared mechanisms of resistance and potential therapeutic interventions.

**Methods:**

We applied a search strategy using four major databases (Medline, PubMed, Embase, and Global Health) to find all known genetic associations identified to date with dengue or West Nile virus disease. Here we present a review of our findings and a meta-analysis of genetic variants identified.

**Results:**

We found genetic variations that are significantly associated with infections of these viruses. In particular we found variation within the OAS1 (meta-OR = 0.83, 95% CI: 0.69–1.00) and CCR5 (meta-OR = 1.29, 95% CI: 1.08–1.53) genes is significantly associated with West Nile virus disease, while variation within MICB (meta-OR = 2.35, 95% CI: 1.68–3.29), PLCE1 (meta-OR = 0.55, 95% CI: 0.42–0.71), MBL2 (meta-OR = 1.54, 95% CI: 1.02–2.31), and IFN-γ (meta-OR = 2.48, 95% CI: 1.30–4.71), is associated with dengue disease.

**Conclusions:**

Despite substantial heterogeneity in populations studied, genes examined, and methodology, significant associations with genetic variants were found across studies within both diseases. These gene associations suggest a key role for immune mechanisms in susceptibility to severe disease. Further research is needed to elucidate the role of these genes in disease pathogenesis and may reveal additional genetic factors associated with disease severity.

**Electronic supplementary material:**

The online version of this article (10.1186/s12879-018-3186-6) contains supplementary material, which is available to authorized users.

## Background

Dengue (DENV) and West Nile (WNV) viruses are mosquito-borne viruses in the *Flaviviridae* family, which also includes other viruses such as Zika and yellow fever. These viruses can cause disease with substantial public health impact. DENV and WNV are found in similar areas of the world, can be carried by the *Aedes* family of mosquitoes, have similar initial stages of infections and similar symptoms of mild febrile illness, and are highly cross-reactive; however, severe disease manifests differently for these two viruses [1–3]. West Nile Virus was first identified in Uganda in 1937, has been endemic in the United States since 1999 [4], and is estimated to have infected 3 million people [5]. While the majority of infections are asymptomatic, ~ 20% of infections lead to mild febrile disease in infected individuals and 1% of infected individual experience severe, neurological disease such as meningitis and encephalitis [6]. DENV has a vastly higher disease burden, with an estimated 50 million cases and 25,000 fatalities worldwide annually [7, 8]. The majority of DENV infections can be classified as asymptomatic or mild febrile illness, with approximately < 1% progressing to Dengue Hemorrhagic Fever (DHF) or Dengue Shock Syndrome (DSS). DHF is delineated from mild DENV febrile illness by the increase in vascular permeability, while DSS has the additional development of circulatory shock [7, 8].

For both WNV and DENV, known risk factors such as immune-compromised states or advanced age are associated with susceptibility to mild and severe disease [9, 10]. The mechanisms by which an infection leads to severe disease in a subset of all infected individuals is incompletely explained. Differing immune responses to infections, including elevated cytokine responses, have been proposed [11–13] and we have recently shown that geographic location is not a driver of severity of WNV infection in a localized region [14]. In addition to similarities in the early stages of infection [15–17], both viruses induce strong immune responses including chemokines (such as IL-8) and cytokines which up-regulate inflammatory reaction (such as TNF-α, IL-1, Il-6, and IFN-β) [18–21]. Renewed interest in understanding flaviviral infection and disease susceptibility comes as climate change expands the number of individuals at risk of exposure to WNV and DENV [3, 22], and with outbreaks of related flaviviruses, most notably Zika [23, 24].

Genetic differences are additional explanations of individual susceptibility to symptomatic disease, and previous genome-wide association studies (GWAS) and candidate-gene studies have identified genetic factors associated with DENV or WNV disease pathogenesis. To assess the current state of knowledge on genetic variation associated with these flaviviral diseases, and to identify any shared features of anti-viral responses, we conducted a systematic review and meta-analysis of the published associations to date between genetic variants and development of DENV or WNV disease.

## Methods

A systematic review of genetic factors and WNV or DENV disease was conducted using the Preferred Reporting Items for Systematic Reviews and Meta-Analyses (PRISMA) (Additional file 1) [25].

### Search strategy

Medline, PubMed, Embase, and Global Health databases were used to search the literature. Search terms included West Nile or DENV and genetic factors; the same set of text words was used for all databases in conjunction with subject headings that were tailored for each database. The text word search specified West Nile or Dengue in the title, a genetic term in the title or abstract, and a human-related term in the title or abstract (Table 1). A sample search strategy is included in the appendix (Additional file 2). Case-control studies which examined at least one genetic factor associated with either viral disease were included. Studies on non-human (e.g., viral, mosquito) genetics and case reports on single patients were excluded. Reports published prior to May 2017 were included in the review. An ancestry search was done of references of selected studies to collect additional potentially relevant references.

**Table 1:** Text word selection for search of selected databases. Text words used for the search strategy, with one term from each column required in the title for the viral term, or in the title or abstract for the genetic and human terms

Viral terms	Genetic Factor terms	Human-related terms
• West Nile • Dengue	▪ microsatellite(s) ▪ genetic variation ▪ genetic factor(s) ▪ genetic marker(s) ▪ genetic analysis/analyses ▪ SNP(s) ▪ single nucleotide polymorphism(s) ▪ copy number variant(s) ▪ genetic predisposition ▪ genetic susceptibility ▪ disease susceptibility ▪ GWAS ▪ genome-wide association study/studies ▪ genetic association(s) ▪ genetic association study/studies ▪ candidate gene study/studies ▪ genetic predisposition to disease ▪ susceptibility to disease ▪ genetic variability ▪ gene identity	▪ human ▪ man/men ▪ woman/women ▪ child/children ▪ teenager(s) ▪ middle-aged ▪ elderly ▪ infant(s) ▪ male(s) ▪ female(s) ▪ patient(s) ▪ participant(s) ▪ citizen(s) ▪ subject(s) ▪ case(s) ▪ control(s)

### Study selection and data extraction

Two researchers reviewed the titles and abstracts of all studies and identified potentially relevant articles within Covidence with 98.6% consistency [26]. Discrepancies were resolved through re-review and mutual consensus. Both researchers read the full text of all of the selected potentially relevant articles and identified the final reports to be included in this review. Data sets were extracted without personal identifiers and organized into literature tables. The main fields included authors, year of publication, country, sample size, case and control group definitions, genotyping method, genes and genetic variants analyzed, genotype count data when available, odds ratios (OR), and statistical analysis method.

When two or more studies examined the same variants, we used the raw genotype data to calculate ORs with 95% confidence intervals using the R package Epitools [27]. When the raw genotype data were not available within the published papers, we requested the data sets from corresponding authors of the studies. Of the six authors contacted, three shared data, two indicated they no longer had access to the data, and one did not respond by date of submission. In order to make comparisons across the different DENV phenotypes used in the studies, we compared asymptomatic DENV infections and controls with all symptomatic infections (DENV fever, DENV hemorrhagic fever, and DENV shock syndrome). Using the genotype data, we calculated ORs for each study under a dominant model, recessive model, homozygote mutant versus homozygote wild-type, and heterozygote versus homozygote wild-type. We meta-analyzed the ORs using RevMan [28]. The genetic model with the most significant meta-OR is presented here. When this model was the homozygote mutant versus homozygote wild or the heterozygote versus homozygote wild, we included both of these models for that particular single nucleotide polymorphism (SNP).

### Quality assessment

We assessed the quality of each study with the Newcastle-Ottawa Quality Assessment Scale for Case-Control Studies [29], which assesses each study’s selection, comparability, and exposure ascertainment approach.

## Results

**Fig. 1:**
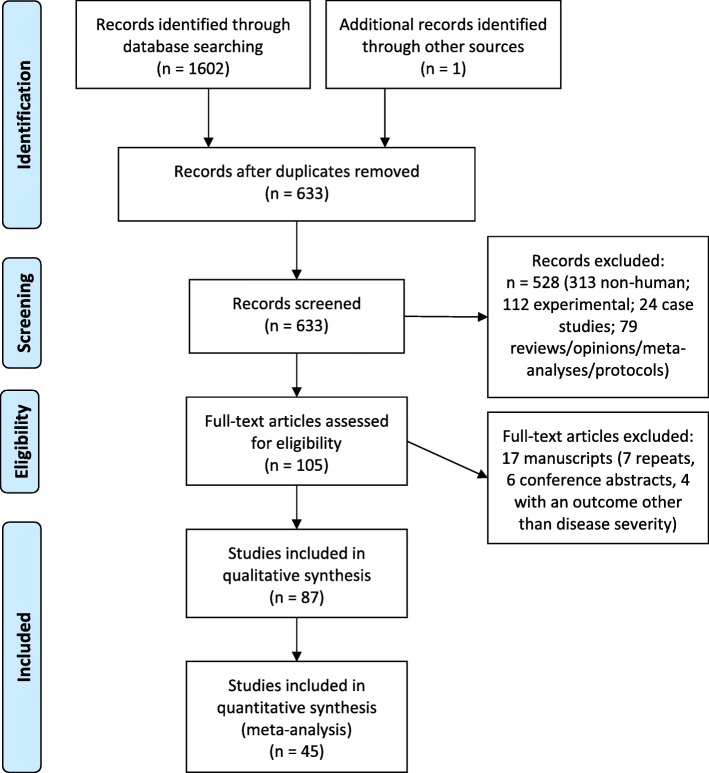
PRISMA Flowchart of strategy to identify papers assessing genetic variation and WNV or DENV disease

To identify all published research assessing the role of genetic variation with DENV or WNV disease, we executed the above search strategy and identified 633 published reports (Table 1, Fig. 1). Two researchers independently reviewed the titles and abstracts of these 633 papers and identified 104 papers for further full-text review in this meta-analysis (Additional file 3). One additional paper from 1987 was identified as pertinent during the ancestry search and was added to the review. The final analysis includes data from 87 of the 105 publications, following exclusion of 18 papers for cause (seven repeats, six conference abstracts, and five with an outcome other than disease severity). Reflecting the higher disease burden and longer research history of DENV virus, of these 87 papers selected, 74 studied DENV-infected populations and 13 focused on WNV.

### HLA genetic variation associated with disease severity

Notably, 27 separate HLA alleles were examined by two or more research groups for an association with severe DENV disease (Additional file 4). Four research groups analyzed HLA alleles for an association with WNV disease (Additional file 5), however there was no overlap in the alleles studied. Although HLA variants show substantial contribution to disease outcome, significant variations in study design, data analysis platforms, data availability and presentation precluded our in-depth meta-analysis of these data.

### Multiple genes are associated with severity of WNV infection

Previous reports of genetic associations with WNV disease severity focused on U.S. or Canadian populations and compared severe and non-severe infections. Overall, these studies identified 12 gene variants and significant findings include SNPs of multiple immune-related genes such as RFC1, SCN1A, and IRF3 (Table 2).

**Table 2:** Genetic variation significantly associated with West Nile virus disease. We include in this table all variants studied by two or more research groups and variants found to have a significant association by one research group

Gene	Genetic variant	Entrez Gene ID [31]	Major Allele	Minor Allele	Number of Cases	Number in Comparison Group	Country	Key Results	Included in meta-analysis
ANPEP	rs25651	290	T	C	560 severe	950 non-severe infections	US & Canada	0.69 odds of severe disease [59]	No^a^
			G	A	39 severe	61 controls	Israel	No significant association with disease [60]	
CACNA1H	rs113802594	8912	A	G	1330 severe	919 non-severe infections	US & Canada	8.58 odds of encephalitis [61]	No
CCR5	CCR5 Δ32	1234	–	Δ32 deletion	560 severe	950 non-Severe infections	US & Canada	No significant association with disease [59]	Yes
			–	Δ32 deletion	39 severe	61 controls	Israel	No significant association with disease [60]	
			–	Δ32 deletion	422 symptomatic	331 asymptomatic infections	US & Canada	No significant association with disease [62]	
			–	Δ32 deletion	634 infections	422 controls	US	No significant association with disease, but significant association with more severe disease (*p* = 0.0016) [46]	
			–	Δ32 deletion	395 symptomatic (two cohorts of 247 and 148)	1318 controls	US	Significantly associated with disease in two cohorts (OR = 4.4 [1.6–11.8] and OR = 9.1 [3.4–24.8]), and fatal outcomes in one cohort with OR = 13.2 [1.9–89.9] [63]	
HERC5	rs148556308	51,191	A	G	1330 severe	919 non-severe infections	US & Canada	Significantly associated with severe disease (*p*-value = 6.5 × 10^− 10^) [61]	No
IRF3	rs2304207	3661	G	C	422 severe	331 asymptomatic infections	US	0.52 odds of symptomatic infection under dominant model [62]	No^a^
			G	C	39 severe	61 controls	Israel	No significant association with disease [60]	
MIF	rs5844572	4282	5 or 6 CATT repeats	7 CATT repeats	518 severe	514 non-severe	US & Canada	1.73 odds of encephalitis among patients with high-expression allele as compared to all other types of WNV disease [64]	No
MX1	rs7280422	4599	C	G	39 severe	61 controls	Israel	4.05 odds of infection associated with variant allele [60]	No^a^
			C	G	422 severe	331 asymptomatic infections	US	0.25 odds of symptomatic infection under a recessive model [62]	
OASL	rs3213545	8638	C	T	422 severe	331 asymptomatic infections	US	No significant association with disease [62]	Yes
			C	T	39 severe	61 controls	Israel	1.85 (1.03–3.3) odds of infection [60]	
			C	T	33 symptomatic	60 controls	US	Significantly associated with disease (*P* < 0.004) [65]	
OAS1	rs10774671	4938	A	G	422 severe	331 asymptomatic infections	US	No significant association with disease [62]	Yes
			A	G	39 severe	61 controls	Israel	No significant association with disease [60]	
			A	G	501 seropositive	552 controls	US	1.6 [95% CI 1.2–2.0] odds of seroconversion [66]	
OAS1	rs34137742	4938	C	T	422 severe	331 asymptomatic infections	US	9.79 [95% CI 3.60–26.61] odds of encephalitis and paralysis [62]	Yes
			C	T	39 severe	61 controls	Israel	No significant association with disease [60]	
RFC1	rs2066786	5981	T	C	560 severe	950 non-severe infections	US & Canada	0.68 odds of severe disease associated with minor allele [59]	No^a^
			G	A	39 severe	61 controls	Israel	2.8 odds under dominant model [60]	
SCN1A	rs2298771	6323	C	T	560 severe	950 non-severe infections	US & Canada	1.47 odds of severe disease associated with minor allele [59]	No^a^
			A	G	39 severe	61 controls	Israel	No significant association with disease [60]	
TFCP2L1	rs11122852	29,842	A	T	1330 severe	919 non-severe infections	US & Canada	3.57 odds of severe disease and 4.94 odds of Acute Flaccid Paralysis than controls [61]	No

^a^genotype data not available for meta-analysis

### OAS1 and CCR5 have significant associations with WNV disease across multiple studies

**Fig. 2:**
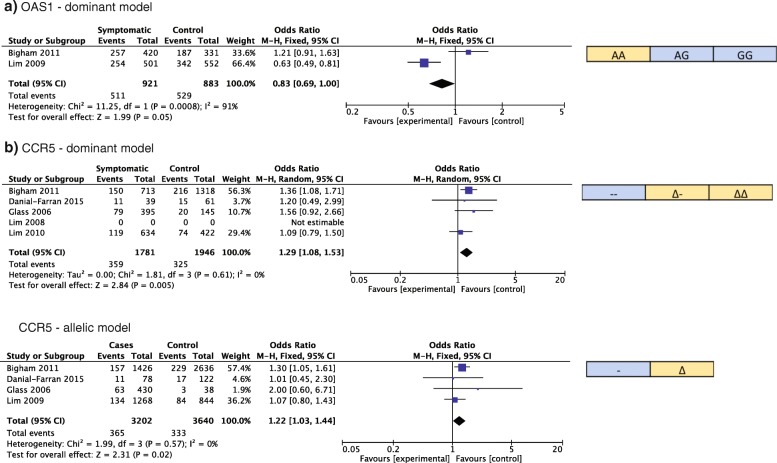
Significant meta-ORs for associations between OAS1 (rs10774671) and CCR5 (Δ32) and West Nile virus disease. Genotype count data from published reports of WNV subjects were meta-analyzed using RevMan. The meta-odds ratio (OR) for more severe disease is indicated with the genetic model for each gene. For each gene, the allele or genotype is shown which is associated with asymptomatic infection and controls (blue) or severe disease (yellow) outcome

For genes with genotype count data available for ≥2 studies, we conducted a meta-analysis of genetic association to disease severity. Meta-analysis allows recognition of well-established genetic associations and identification of redundant studies for genes with null associations. We found that SNPs in MX1, OASL, OAS1, RFC1, and CCR5 were studied by multiple research groups for an association with WNV disease (Table 2). To assess the overall association of these SNPs with WNV disease, we calculated a combined OR for each gene based on the genotype counts under four different genetic models. Of these, CCR5 and OAS1 meta-ORs were significant under a dominant model, with meta-OR of 0.83 [95% CI: 0.69–1.00] and 1.29 [1.08–1.53], respectively (Fig. 2). The CCR5 meta-OR was also significant under an allelic model with a meta-OR of 1.22 [95% CI: 1.03–1.44]. The CCR5 delta 32 deletion is associated with more severe disease while the OAS1 allele G was associated with less severe disease.

### Multiple genes are associated with severity of DENV infection

Seventy-four studies have examined genetic associations with DENV disease severity and more than 30 genes have been implicated in DENV disease (Additional file 3). SNPs that were studied by only a single research group are presented in Table 3. We also include SNPs studied by multiple research groups, but for which genotype data was unavailable or the comparison groups of multiple studies could not be analyzed together.

**Table 3:** Genetic variation associated with DENV disease. We include in this table all variants studied by two or more research groups and variants found to have a significant association by one research group

Gene	Genetic Variation	Entrez Gene ID [31]	Major Allele	Minor Allele	Number of Cases	Number in Comparison Group	Country	Key Results	Meta-Analyzed
BAK1	rs5745568	578	G	T	509 DHF/DSS	409 DF	Thailand	1.32 (1.09–1.60) odds of severe disease associated with G allele [67]	No
CCR5	rs333	1234	–	Δ32 deletion	56 DF	91 controls	Australia	No significant association with disease [68]	Yes
					88 DHF/DSS	335 controls	Brazil	No significant association with disease [69]	
CD209/ DCSIGN	rs480803	30,835	A	G	509 DHF/DSS	409 DF	Thailand	No significant association with disease [67]	Yes
			A	G	88 DHF/DSS	335 controls	Brazil	No significant association with disease [69]	
			A	G	112 symptomatic	104 controls	India	No significant association with disease [70]	
			A	G	103 symptomatic	145 asymptomatic infections	Mexico	No significant association with disease [71]	
			A	G	156 DF and 12 DHF	72 controls	Brazil	No significant association with disease [72]	
			A	G	606 symptomatic	696 controls	Thailand	5.84 (2.77–12.31) odds of DHF compared to DF and 0.204 (*P* = 2.0 × 10^− 6^) odds of symptomatic infection compared to controls [73]	
			A	G	286 symptomatic	236 asymptomatic infections	Brazil	No significant association with disease [74]	
			A	G	176 DF and 135 DH0046	120 controls	Taiwan	2.36 (1.12–4.97) odds of symptomatic infection compared to controls, 3.68 (1.67–8.09) odds of DHF compared to controls, and 2.46 (1.32–4.59) odds of DHF compared to DF [75]	
CFH	rs3753394	3075	C	T	187 DHF	121 DF	Thailand	No significant association with disease [76]	No^a^
			C	T	87 DHF	34 DF	Brazil	2.53 (1.38–4.69) odds of severe disease compared to mild disease under dominant model [77]	
CLEC5A	rs1285933	23,601	T	C	88 DHF/DSS	335 controls	Brazil	2.25 (1.07–4.87) odds of severe disease for TT compared to CC genotype [69]	No
CXCL8/ IL8	rs4973	3576	T	A	45 DHF	108 controls	India	0.43 (0.20–0.93) odds of severe disease [78]	No
DDX58	rs3205166	23,586	T	G	120 DENV positive	109 controls	India	0.66 odds of disease associated with G allele for rs3205166 [79]	No
	rs11795343								
	rs669260								
FCγRII-α	rs1801274	2212	A (H amino acid)	G (R amino acid)	103 symptomatic	145 asymptomatic infections	Mexico	0.51 (0.26–0.98) odds of symptomatic infection and 0.45 (0.21–0.96) odds of severe disease compared to controls [71]	Yes
			T (H)	C (R)	89 DF and 33 DHF	107 controls	India	No significant association with disease [80]	
			T (H)	C (R)	68 DF, 29 DHF/DSS	42 asymptomatic infections	Cuba	10.56 (2.33–54.64) odds of DHF compared to asymptomatic disease for under dominant model [81]	
			T (H)	C (R)	302 DHF	238 controls	Vietnam	No significant association with disease [82]	
			T (H)	C (R)	40 DF, 30 DHF/DSS	40 asymptomatic infections	Pakistan	3.21 (1.29–7.97) odds of symptomatic disease, 2.82 (1.00–7.97) odds of DF, and 3.90 (1.13–13.07) odds of DHF/DSS over asymptomatic infection [83]	
HPA	HPA 1a/1a	Not available	1a antigen	1b antigen	75 DHF	90 DF	India	1.93 odds (*p* = 0.006) of severe disease [84]	No
	HPA 2a/2b		2a antigen	2b antigen	75 DHF	90 DF	India	2.8 odds (*p* = 0.007) of severe disease [84]	No
IFN-γ	rs2430561	3458	A	T	80 symptomatic	100 DEN-negative febrile cases and 99 healthy controls	Brazil	2.23 (*p* = 0.0255) odds compared to DEN-negative and 2.37 (*p* = 0.0165) odds compared to controls [85]	Yes
			A	T	25 DHF	41 DF	Venezuela	No significant association with disease [86]	
			A	T	43 DHF	99 controls	Cuba	No significant association with disease [87]	
IL-1B	rs16944	3553	A	G	45 DHF	108 controls	India	No significant association with disease [78]	Yes
			C	T	118 symptomatic	80 controls	Brazil	No significant association with disease [88]	
	rs1143627		C	T	367 secondary DHF and 74 secondary DSS	313 secondary DF	Thailand	3.49 (1.36–8.95) odds of DSS compared to DHF and 2.81 (1.12–7.06) odds of DSS compared to DF under dominant models [89]	No
IL-1RA	86 base pair tandem repeat	3557	4 repeats	2 repeats	367 secondary DHF and 74 secondary DSS	313 secondary DF	Thailand	1.86 (1.05–3.26) odds of DSS compared to DHF and 1.86 (1.05–3.27) odds of DSS compared to DF for the 2/4 genotype [89]	No^a^
			1 repeat	2–4 repeats	280 DHF	229 controls	Vietnam	No significant association between IL-1RA repeats and DHF [82]	
IL-6	rs1800795	3569	G	C	25 DHF	41 DF	Venezuela	No significant association with disease [86]	Yes
			G	C	43 DHF	99 controls	Cuba	No significant association with disease [87]	
			G	C	118 symptomatic	80 controls	Brazil	No significant association with disease [88]	
			G	C	200 DF	309 controls	Brazil	0.62 (0.42–0.91) odds of disease among heterozygotes compared to homozygote wild-type [90]	
IL-10	rs1800871	3586	C	T	88 DSS	335 controls	Brazil	No significant association with disease [69]	Yes
			A	G	45 DHF	108 controls	India	No significant association with disease [78]	
			C	T	25 DHF	41 DF	Venezuela	No significant association with disease [86]	
			C	T	43 DHF	99 controls	Cuba	No significant association with disease [87]	
			C	T	200 DF	309 controls	Brazil	No significant association with disease [90]	
			T	C	86 DF, 182 DHF, 14 DSS	120 controls	Malaysia	No significant association with disease [91]	
			C	T	107 DHF	62 controls	Sri Lanka	No significant association with disease [92]	
	rs1800872		C	A	25 DHF	41 DF	Venezuela	No significant association with disease [86]	Yes
			C	A	43 DHF	99 controls	Cuba	No significant association with disease [87]	
			C	A	200 DF	309 controls	Brazil	No significant association with disease [90]	
			A	C	86 DF, 182 DHF, 14 DSS	120 controls	Malaysia	No significant association with disease [91]	
			A	C	107 DHF	62 controls	Sri Lanka	No significant association with disease [92]	
	rs1800896		A	G	25 DHF	41 DF	Venezuela	No significant association with disease [86]	Yes
			A	G	43 DHF	99 controls	Cuba	No significant association with disease [87]	
			A	G	200 DF	309 controls	Brazil	No significant association with disease [90]	
			A	G	86 DF, 182 DHF, 14 DSS	120 controls	Malaysia	No significant association with disease [91]	
			A	G	107 DHF	62 controls	Sri Lanka	No significant association with disease [92]	
JAK1	rs11208534	3716	T	C	50 DHF	236 DF	Brazil	4.20 (1.7–10.4) odds of severe disease [74]	No
	rs2780831		G	A	50 DHF	236 DF	Brazil	2.1 (1.1–4.1) odds of severe disease [74]	No
	rs310196		T	G	50 DHF	236 DF	Brazil	0.4 (0.2–0.7) odds of severe disease [74]	No
MBL2	Exon 1	4153	A	O	110 symptomatic	150 controls	Brazil	No significant association with disease [93]	Yes
			A	O	57 DHF	104 DF	Brazil	7.24 (1.38–38.02) odds of DHF among OO genotype compared to AA genotype [94]	
MICB	rs3132468	4277	T	C	76 DSS	409 DF, 432 DHF	Thailand	1.58 (1.02–2.40) odds of DSS compared to non-DSS [95]	Yes
			T	C	2008 DSS	2018 controls	Vietnam	1.34 (1.23–1.46) odds of DSS per allele [96]	
			T	C	3961 cases	1068 controls	Vietnam	1.42 (1.20–1.64) odds of DSS per allele [97]	
PLCE1	rs3740360	51,196	A	C	2008 DSS	2018 controls	Vietnam	0.80 (0.75–0.86) odds per allele of DSS [96]	Yes
			A	C	3961 cases	1068 controls	Vietnam	0.77 (0.59–0.99) odds per allele of DSS [97]	
	rs3765524		C	T	76 DSS	409 DF, 432 DHF	Thailand	1.49 (1.00–2.26) odds of DSS compared to non-DSS [95]	Yes
			C	T	2008 DSS	2018 controls	Vietnam	0.80 (0.75–0.86) odds per allele of DSS [96]	
RXRA	rs12339163	6256	G	A	60 DHF	137 asymptomatic infections and controls	Cuba	0.36 (0.17–0.77) odds of severe disease [98]	No
	rs3118593		A	C	60 DHF	137 asymptomatic infections and controls	Cuba	0.44 (0.25–0.77) odds of severe disease [98]	
	rs4262378		G	A	60 DHF	137 asymptomatic infections and controls	Cuba	0.41 (0.24–0.72) odds of severe disease [98]	
	rs4424343		A	G	60 DHF	137 asymptomatic infections and controls	Cuba	0.43 [0.24–0.76] odds of severe disease [98]	
	rs62576287		C	A	60 DHF	137 asymptomatic infections and controls	Cuba	0.10 (0.01–0.83) odds of severe disease [98]	
TAP1	amino acid 333	6890	Ile	Val	90 DF, 75 DHF, 32 DSS	100 controls	India	2.58 (p = 0.007) odds of DHF among heterozygotes compared to DF [84]	Yes
			Ile	Val	107 DHF	62 controls	Sri Lanka	No significant association with disease [92]	
TAP2	amino acid 379	6891	Val	Ile	107 DHF	62 controls	Sri Lanka	No significant association with disease [92]	Yes
			Val	Ile	90 DF, 75 DHF, 32 DSS	100 controls	India	2.11 (p = 0.001) odds of DHF among heterozygotes [99]	
TGFβ1	rs1800471	7040	G	C	25 DHF	41 DF	Venezuela	No significant association with disease [86]	
			G	C	200 DF	309 controls	Brazil	No significant association with disease [90]	
	rs1982073		T	C	25 DHF	41 DF	Venezuela	No significant association with disease [86]	Yes
			T	C	43 DHF	99 controls	Cuba	No significant association with disease [87]	
			T	C	200 DF	309 controls	Brazil	No significant association with disease [90]	
TIRAP	rs8177374	114,609	C	T	33 DHF	109 controls	India	2.64 (1.17–5.99) odds of severe disease among heterozygotes [100]	No
TLR3	rs3775291	7098	C	T	33 DHF	87 DF	India	0.39 (0.16–0.88) odds of severe disease associated with T allele [100]	No
TNF-α	rs361525	7124	G	A	86 DF, 182 DHF, 14 DSS	120 controls	Malaysia	4.92 (1.10–21.90) odds of DHF compared to control group for heterozygotes [91]	Yes
			G	A	41 DF, 32 DHF	169 controls	Mexico	0.19 (0.02–0.78) odds of disease with A allele [101]	
	rs1800629		G	A	80 symptomatic	100 DEN-negative febrile cases and 99 healthy controls	Brazil	No significant association with disease [85]	Yes
			G	A	25 DHF	41 DF	Venezuela	2.5 (1.47–4.13) odds of severe disease [86]	
			G	A	43 DHF	99 controls	Cuba	3.51 (1.77–7.00) odds of severe disease [87]	
			G	A	200 DF	309 controls	Brazil	No significant association with disease [90]	
			G	A	86 DF, 182 DHF, 14 DSS	120 controls	Malaysia	0.43 (0.22–0.84) odds of DHF compared to control for heterozygotes [91]	
			G	A	107 DHF	62 controls	Sri Lanka	2.53 (1.10–5.83) odds of disease for GG genotype [92]	
			G	A	85 DF, 29 DHF	110 controls	India	No significant association with disease [102]	
			G	A	19 DF, 82 DHF	106 controls	Thailand	No significant association with disease [103]	
			G	A	85 DF, 45 DHF	163 controls	Mexico	No significant association with disease [101]	
TLR4	amino acids 299 and 399	7099	Asp299, Thr399	Gly299, Ile399	201 DHF	179 controls	Indonesia	No significant association with disease [104]	Yes
			Asp299, Thr399	Gly299, Ile399	63 DF, 57 DHF/DSS	200 controls	India	2.00 (1.17–3.43) odds associated with Gly299 for DF versus controls, and 2.38 (1.16–4.85) associated with Ile399 for DF versus controls [105]	
VDR	rs731236	7421	T	C	302 DHF	238 controls	Vietnam	Associated with more severe disease (*p* = 0.033) [82]	Yes
			T	C	83 DF, 29 DHF	105 controls	India	No significant association with disease [106]	

^a^ genotype data not available for meta-analysis

### Significant associations with DENV disease

**Fig. 3:**
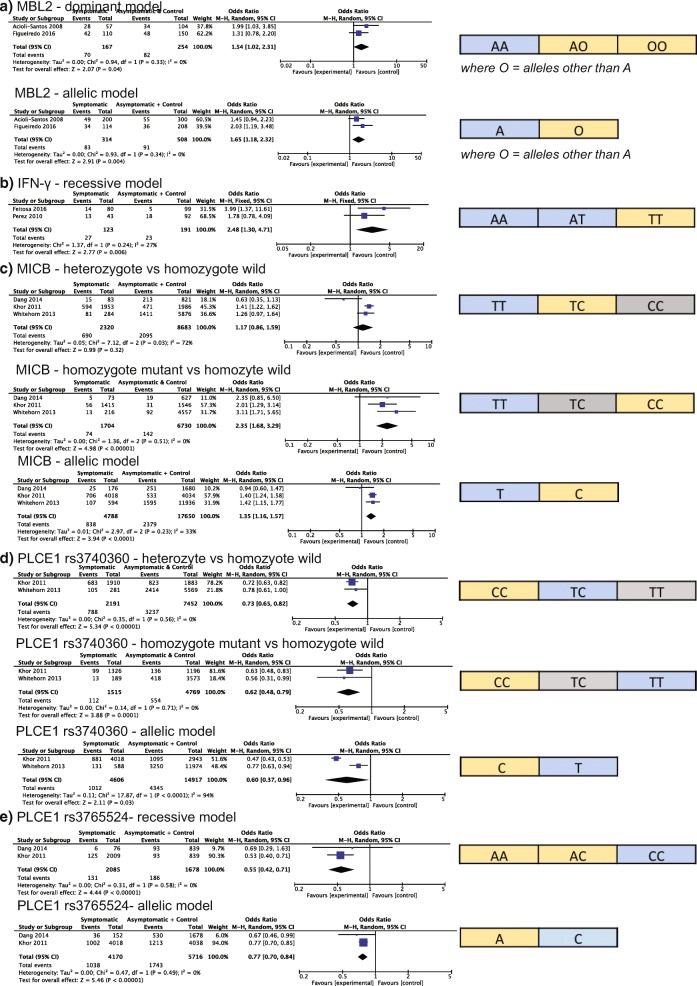
Meta-analyzed genetic variation associated with DENV disease. Genotype count data from published reports of WNV subjects were meta-analyzed using RevMan. The meta-odds ratio (OR) for more severe disease is indicated with the genetic model for each gene: MBL2 (**a**), IFN-γ (**b**), MICB (**c**), PLCE1 (**d** and **e**). If multiple models were significant, we present the most significant model. The alleles or genotypes associated with asymptomatic infection and controls (blue) or with severe disease (yellow) outcome are shown for each gene

Among the DENV studies, the same variant within 17 genes was studied by two or more research groups (Table 3). Four genes had significant meta-ORs (Fig. 3). For a SNP in MBL2 (exon 1), we calculated a meta-OR of 1.54 [1.02–2.31] under a dominant model and 1.65 [1.18–2.32] under an allelic model, with alleles other than the A allele being associated with more severe disease. The T allele for SNP rs2430561 in the IFN-γ gene was associated with severe disease under a recessive model with a meta-OR of 2.48 [0.30–4.71]. For a SNP located within MICB (rs3132468), we found the CC genotype had a significantly greater association with severe disease (meta-OR 2.35 [1.68–3.29]), but the heterozygote genotype showed no significant association with disease severity as compared to the TT genotype (meta-OR = 1.17 [0.86–1.59]). For this SNP, the C allele was also found to be significantly associated with disease as compared to the T allele (meta-OR = 1.35 [1.16–1.57]). For two SNPs located within the PLCE1 gene, every model tested was significant, with the most significant meta-ORs being 0.62 [0.48–0.79] for TT genotype as compared to CC genotype for rs3740360 and 0.55 [0.42–0.71] under a recessive model for rs3765524. TNF-α (rs1800629 and rs361525) was the most studied gene, but none of the models tested provided a significant meta-OR.

### Quality scores

Based on the Newcastle Ottawa Scoring System, the average quality score was 5.76 (range: 3–7) for the WNV publications and 5.10 (range: 2–7) for the DENV publications (Additional file 6). We also assessed whether the study authors corrected for multiple testing, and found less than half of both WNV and DENV studies provided corrected *p*-values when appropriate, indicating an inflated type I error rate.

## Discussion

We have examined genetic variants that show association with DENV or WNV disease severity. This analysis was undertaken to identify genetic differences that are significant drivers of susceptibility to symptomatic disease that may shed light on mechanisms of immune resistance to these viruses. Among the 87 studies examined, a wide range of genetic targets was found to be significant, with many of the genes unsurprisingly playing a key role in the immune system defense against viral infections (Additional file 7).

Despite the large number of studies, only 27 genes were studied by more than one research group for an association with either disease. Throughout these studies, several key genes rose to the forefront as the most studied and the most significant associations. Many studies focused on the HLA region of the genome, and, although inconsistencies in data presentation preclude a meta-analyze of these results, there were clear signs of the importance of this area for both diseases.

With the central role of HLA for the immune system, polymorphisms in this region have been well studied for associations with disease. The area is highly polymorphic, however, leading to difficulties for comparing the diverse range of alleles. Adding to this complexity, DENV serotypes interact differently with HLA [30]. The regions identified in this systematic review, including DRB1, DQA1, DQB1, A, B, and C, are among the most diverse regions of the HLA region [31]. A recent study examined some of these regions by supertype, and found the B44 supertype could be protective against DHF during secondary infections and that the A02 and A01/03 supertypes could be associated with more severe disease [32].

KIR genes, which are expressed on the surface of natural killer cells, also have wide genetic variability as noted with HLA genes [33]. While several KIR alleles were studied in DENV-infected populations, only one publication to date has examined KIR genotypes in West Nile virus-infected individuals, and this study had a sample size of four [34]. The results suggested a possible association; this, in conjunction with the results of the DENV research in this area and the genes’ highly polymorphic nature, could be an area that should be explored further. Infection with WNV has been shown to lead to diversification of KIR receptor expression [35]. In addition to the research outlined above, researchers have examined the association of KIR genotypes with DENV infection in vitro. Within the in vitro research, the timing of natural killer cell activation has been linked to disease severity and interactions between KIR and HLA have been suggested [36–38].

Another key non-HLA gene identified to be associated with WNV disease was OASL, which codes for an enzyme that is induced by type 1 interferon and viruses [39]. OASL was first identified to have a potentially critical role in WNV disease pathogenesis in 2002, when researchers found that mice with a truncated form of the gene were more susceptible to disease [40]. Elevated activity of the OAS genes has also been associated with more severe DENV infection in vitro [41]. This data and the significance of variation within the OAS genes for WNV outcomes highlight the importance of the interferon pathways in response to flavivirus infections and suggest a need for further in depth examination the association of genetic variability within OAS and DENV severity.

CCR5Δ32 was the only gene studied by two or more research groups for each disease. CCR5 was first identified as a co-receptor for HIV in 1996, and CCR5 deficiency, or a homozygous genotype of CCR5Δ32, was found to be protective against HIV infection [42–45]. In West Nile, CCR5 deficiency is not associated with incidence of infection, but is associated with severity of disease for infected individuals [46]. Subsequent research showed that CCR5 specifically plays a role in the ability of cortical neurons to combat West Nile virus infection of the brain [47]. In DENV, CCR5 deficiency has been linked with increased viral load and disease severity [48]. The study also found that the CCR5 receptor in macrophages is necessary for replication of DENV serotype 2, an early step in the infection process [49]. Given the similarities of these flaviviral diseases and the significant association of CCR5, the only gene looked at by research groups for both diseases, further research could be beneficial to further understanding the role of genetic variation in the development of severe flavivviral disease [50].

When we meta-analyzed the DENV studies, we found significant associations between DENV disease and genetic variation in MBL2, PLCE1, IFN**-**γ, and MICB. The role of many of these genes in disease pathogenesis has been characterized through in vivo and in vitro studies. MBL2, the mannose-binding lectin 2 gene, encodes a protein with a role in innate immunity and complement pathway, while PLCE1 encodes an enzyme critical to the generation of the inositol 1,4,5-triphosphate (IP3) and diacylglycerol (DAG) messengers [51]. MICB and IFN-y are both critical in the immune response, and thus variations within these genes could have strong effects on the initial response to the viral infection and the subsequent disease pathogenesis [51].

Our study is limited by several factors, most notably by the available literature. To ensure we found as many papers as possible, we constructed a search strategy that involved multiple databases, used both subject headings and text words, was not limited to English articles, and included an ancestry search [52]. Despite our focus on significant results and genes studied by at least two research groups, the wide heterogeneity among the populations studied limited our ability to interpret the meta-analyzed results. Lack of diversity in genetic studies is well-documented [53], and the absence of certain affected populations, particularly in Africa, among the identified studies further demonstrates this unmet research need [54]. The diversity of results among studies that examined the same SNP could be due to population heterogeneity, as well as to differences in study approach, including selection of control and comparison groups. Additionally, previous exposure history, DENV serotype, and WNV or DENV genotype are all factors that can affect disease severity, but were not accounted for in the included studies [4]. The number and type of genes examined varied greatly between studies, and we were limited by what genes researchers chose to sequence and include in publications. The unavailability of comparable genotype data and the incomparability of research groups across some studies preclude a more in depth analysis at present.

## Conclusions

The genes found to be significantly associated with WNV or DENV disease pathogenesis varied in function, with most being linked to the immune response. As the regions of the world affected by WNV, DENV, and related viruses such as Zika, continue to expand due in part to climate change, an improved understanding of the association between genetic variation and disease severity will be valuable for all potentially affected populations [55–58]. Based on the growing incidence of these diseases, the paucity of consistency in the associations found, and the limited overlap in genetic targets studied to date, there is need for continued and deeper studies examining the role of genetic factors in WNV and DENV disease severity. In addition to conducting new studies such as whole-exome sequencing within larger population samples, further analyses could be conducted of existing data, to glean novel findings such as gene-gene or gene-environment interactions, rare and low frequency variants, and pathways of significant determinants of anti-viral resistance.

## Additional files

Additional file 1: PRISMA Checklist. This systematic review of genetic factors and WNV or dengue disease was conducted using the Preferred Reporting Items for Systematic Reviews and Meta-Analyses (PRISMA) guidelines outlined in this checklist. (DOC 95 kb)

Additional file 2: Sample Search Strategy for the Embase Database. Medline, PubMed, Embase, and Global Health databases were used to search the literature. Search terms included West Nile or Dengue and genetic factors; the same set of text words was used for all databases in conjunction with subject headings that were tailored for each database. As an example, this search strategy for the Embase database is provided. These subject headings, in conjunction with the text search (Table 1), were used to find relevant literature in Embase. (DOCX 13 kb)

Additional file 3: Included Papers. All publications included in this review are listed, with study details on authors, year of publication, country, sample size, case and control group definitions, and genotyping method. (XLSX 54 kb)

Additional file 4: HLA Associations with DENV Disease Severity. HLA alleles studied by two or more research groups for association with DENV disease severity. (DOCX 311 kb)

Additional file 5: HLA Associations with WNV Disease Severity. All examined targets from the analyzed publications are listed, with significant associations shown in bold. (XLSX 9 kb)

Additional file 6: Quality Scores. The quality of each study was evaluated with the Newcastle-Ottawa Quality Assessment Scale for Case-Control Studies, which assesses each study’s selection, comparability, and exposure ascertainment approach. (XLSX 16 kb)

Additional file 7: Extracted Data from Included Studies. All genotype data extracted from the manuscripts or collected from study authors is provided. (XLSX 54 kb)

## Abbreviations

DENV: Dengue virus
DHF: Dengue Hemorrhagic Fever
DSS: Dengue Shock Syndrome
GWAS: genome-wide association studies
PRISMA: Preferred Reporting Items for Systematic Reviews and Meta-Analyses
SNP: single nucleotide polymorphism
WNV: West Nile virus
