# Supplementary material for: Identification of genetic variants associated with dengue or West Nile virus disease: a systematic review and meta-analysis
Source: BMC Infect Dis. 2018 Jun 22;18:282. doi: 10.1186/s12879-018-3186-6 (PMC6014009; doi:10.1186/s12879-018-3186-6)
Supplement: Supplementary file 1 — PRISMA Checklist. This systematic review of genetic factors and WNV or dengue disease was conducted using the Preferred Reporting Items for Systematic Reviews and Meta-Analyses (PRISMA) guidelines outlined in this checklist. (DOC 95 kb) [file 12879_2018_3186_MOESM1_ESM.doc]

| **Section/topic** | **#** | **Checklist item** | **Reported on page #** |
| --- | --- | --- | --- |
| **TITLE** | | |  |
| Title | 1 | Identify the report as a systematic review, meta-analysis, or both. | Lines 1-2: **“Genetic Variants Associated with Dengue or West Nile Virus Disease: a Literature Review and Meta-Analysis”** |
| **ABSTRACT** | | |  |
| Structured summary | 2 | Provide a structured summary including, as applicable: background; objectives; data sources; study eligibility criteria, participants, and interventions; study appraisal and synthesis methods; results; limitations; conclusions and implications of key findings; systematic review registration number. | Background – Line 13 – 20: “Dengue and West Nile viruses are highly cross-reactive and have numerous parallels in geography, vector host (*Aedes* family of mosquitoes), and initial symptoms of infection. While the vast majority (>80%) of both dengue and West Nile virus infections result in asymptomatic infections, a minority of individuals experience symptomatic infection and an even smaller proportion develop severe disease. The mechanisms by which these infections lead to severe disease in a subset of infected individuals is incompletely understood, but individual host differences including genetic factors and immune responses have been proposed. We sought to identify genetic risk factors that are associated with more severe disease outcomes for both viruses in order to shed light on mechanisms of resistance and potential therapeutic interventions.”  Objective – Line 20 – 22: “We sought to identify genetic risk factors that are associated with more severe disease outcomes for both viruses in order to shed light on mechanisms of resistance and potential therapeutic interventions.”  Data Sources/Criteria – Lines 23-25: “We applied a search strategy using four major databases (Medline, PubMed, Embase, and Global Health) to find all known genetic associations identified to date with dengue or West Nile virus disease”  Results – Lines 28 – 33: “We found genetic variations that are significantly associated with infections of these viruses. In particular we found variation within the OAS1 (meta-OR = 0.83, 95% CI: 0.69 – 1.00) and CCR5 (meta-OR = 1.29, 95% CI: 1.08 – 1.53) genes is significantly associated with West Nile virus disease, while variation within MICB (meta-OR = 2.35, 95% CI: 1.68 – 3.29), PLCE1 (meta-OR = 0.55, 95% CI: 0.42 – 0.71), MBL2 (meta-OR = 1.54, 95% CI: 1.02 – 2.31), and IFN-γ (meta-OR = 2.48, 95% CI: 1.30 – 4.71), is associated with dengue disease.”  Limitations – Lines 34 – 35: “Despite substantial heterogeneity in populations studied, genes examined, and methodology, significant associations with genetic variants were found across studies within both diseases.”  Implications – Lines 36 – 39: “These gene associations suggest a key role for immune mechanisms in susceptibility to severe disease. Further research is needed to elucidate the role of these genes in disease pathogenesis and may reveal additional genetic factors associated with disease severity.”  Registration Number – N/A |
| **INTRODUCTION** | | |  |
| Rationale | 3 | Describe the rationale for the review in the context of what is already known. | Lines 88 – 91: “Genetic differences are additional explanations of individual susceptibility to symptomatic disease, and previous genome-wide association studies (GWAS) and candidate-gene studies have identified genetic factors associated with dengue or WNV disease pathogenesis.” |
| Objectives | 4 | Provide an explicit statement of questions being addressed with reference to participants, interventions, comparisons, outcomes, and study design (PICOS). | Lines 91 – 95: “To assess the current state of knowledge on genetic variation associated with these flaviviral diseases, and to identify any shared features of anti-viral responses, we conducted a systematic review and meta-analysis of the published associations to date between genetic variants and development of dengue or WNV disease.” |
| **METHODS** | | |  |
| Protocol and registration | 5 | Indicate if a review protocol exists, if and where it can be accessed (e.g., Web address), and, if available, provide registration information including registration number. | N/A |
| Eligibility criteria | 6 | Specify study characteristics (e.g., PICOS, length of follow-up) and report characteristics (e.g., years considered, language, publication status) used as criteria for eligibility, giving rationale. | Lines 103 – 112: “Search terms included West Nile or Dengue and genetic factors; the same set of text words was used for all databases in conjunction with subject headings that were tailored for each database. The text word search specified West Nile or Dengue in the title, a genetic term in the title or abstract, and a human-related term in the title or abstract (Table 1). A sample search strategy is included in the appendix (Table S1). Case-control studies which examined at least one genetic factor associated with either viral disease were included. Studies on non-human (e.g., viral, mosquito) genetics and case reports on single patients were excluded. Reports published prior to May 2017 were included in the review. An ancestry search was done of references of selected studies to collect additional potentially relevant references.” |
| Information sources | 7 | Describe all information sources (e.g., databases with dates of coverage, contact with study authors to identify additional studies) in the search and date last searched. | Line 102: “Medline, PubMed, Embase, and Global Health databases were used to search the literature.”  Lines 130 – 133: “When the raw genotype data were not available within the published papers, we requested the data sets from corresponding authors of the studies. Of the six authors contacted, three shared data, two indicated they no longer had access to the data, and one did not respond by date of submission.”  Line 110 – 112: “Reports published prior to May 2017 were included in the review. An ancestry search was done of references of selected studies to collect additional potentially relevant references.” |
| Search | 8 | Present full electronic search strategy for at least one database, including any limits used, such that it could be repeated. | Supplemental Table 1. Embase Search Strategy |
| Study selection | 9 | State the process for selecting studies (i.e., screening, eligibility, included in systematic review, and, if applicable, included in the meta-analysis). | Lines 119 – 123: “Two researchers reviewed the titles and abstracts of all studies and identified potentially relevant articles within Covidence with 98.6% consistency [27]. Discrepancies were resolved through re-review and mutual consensus. Both researchers read the full text of all of the selected potentially relevant articles and identified the final reports to be included in this review.”  Line 128 – 131: “When two or more studies examined the same variants, we used the raw genotype data to calculate ORs with 95% confidence intervals using the R package Epitools [28]. When the raw genotype data were not available within the published papers, we requested the data sets from corresponding authors of the studies.” |
| Data collection process | 10 | Describe method of data extraction from reports (e.g., piloted forms, independently, in duplicate) and any processes for obtaining and confirming data from investigators. | Lines 123 – 127: “Data sets were extracted without personal identifiers and organized into literature tables. The main fields included authors, year of publication, country, sample size, case and control group definitions, genotyping method, genes and genetic variants analyzed, genotype count data when available, odds ratios (OR), and statistical analysis method.”  Lines 130 – 133: “When the raw genotype data were not available within the published papers, we requested the data sets from corresponding authors of the studies. Of the six authors contacted, three shared data, two indicated they no longer had access to the data, and one did not respond by date of submission.” |
| Data items | 11 | List and define all variables for which data were sought (e.g., PICOS, funding sources) and any assumptions and simplifications made. | Lines 103 – 107: “Search terms included West Nile or Dengue and genetic factors; the same set of text words was used for all databases in conjunction with subject headings that were tailored for each database. The text word search specified West Nile or Dengue in the title, a genetic term in the title or abstract, and a human-related term in the title or abstract (Table 1). A sample search strategy is included in the appendix (Table S1).” |
| Risk of bias in individual studies | 12 | Describe methods used for assessing risk of bias of individual studies (including specification of whether this was done at the study or outcome level), and how this information is to be used in any data synthesis. | Lines 143 – 146: “We assessed the quality of each study with the Newcastle-Ottawa Quality Assessment Scale for Case-Control Studies [30], which assesses each study’s selection, comparability, and exposure ascertainment approach.” |
| Summary measures | 13 | State the principal summary measures (e.g., risk ratio, difference in means). | Lines 136 – 140: “Using the genotype data, we calculated ORs for each study under a dominant model, recessive model, homozygote mutant versus homozygote wild-type, and heterozygote versus homozygote wild-type. We meta-analyzed the ORs using RevMan [29]. The genetic model with the most significant meta-OR is presented here.” |
| Synthesis of results | 14 | Describe the methods of handling data and combining results of studies, if done, including measures of consistency (e.g., I^2^) for each meta-analysis. | Line 128 – 142: “When two or more studies examined the same variants, we used the raw genotype data to calculate ORs with 95% confidence intervals using the R package Epitools [28]. When the raw genotype data were not available within the published papers, we requested the data sets from corresponding authors of the studies. Of the six authors contacted, three shared data, two indicated they no longer had access to the data, and one did not respond by date of submission. In order to make comparisons across the different dengue phenotypes used in the studies, we compared asymptomatic dengue infections and controls with all symptomatic infections (dengue fever, dengue hemorrhagic fever, and dengue shock syndrome). Using the genotype data, we calculated ORs for each study under a dominant model, recessive model, homozygote mutant versus homozygote wild-type, and heterozygote versus homozygote wild-type. We meta-analyzed the ORs using RevMan [29]. The genetic model with the most significant meta-OR is presented here. When this model was the homozygote mutant versus homozygote wild or the heterozygote versus homozygote wild, we included both of these models for that particular SNP.”  Figures 2 and 3 |

Page 1 of 2

| **Section/topic** | **#** | **Checklist item** | **Reported on page #** |
| --- | --- | --- | --- |
| Risk of bias across studies | 15 | Specify any assessment of risk of bias that may affect the cumulative evidence (e.g., publication bias, selective reporting within studies). | Line 128 – 142: “When two or more studies examined the same variants, we used the raw genotype data to calculate ORs with 95% confidence intervals using the R package Epitools [28]. When the raw genotype data were not available within the published papers, we requested the data sets from corresponding authors of the studies. Of the six authors contacted, three shared data, two indicated they no longer had access to the data, and one did not respond by date of submission. In order to make comparisons across the different dengue phenotypes used in the studies, we compared asymptomatic dengue infections and controls with all symptomatic infections (dengue fever, dengue hemorrhagic fever, and dengue shock syndrome). Using the genotype data, we calculated ORs for each study under a dominant model, recessive model, homozygote mutant versus homozygote wild-type, and heterozygote versus homozygote wild-type. We meta-analyzed the ORs using RevMan [29]. The genetic model with the most significant meta-OR is presented here. When this model was the homozygote mutant versus homozygote wild or the heterozygote versus homozygote wild, we included both of these models for that particular SNP.”  Figures 2 and 3 |
| Additional analyses | 16 | Describe methods of additional analyses (e.g., sensitivity or subgroup analyses, meta-regression), if done, indicating which were pre-specified. | N/A |
| **RESULTS** | | |  |
| Study selection | 17 | Give numbers of studies screened, assessed for eligibility, and included in the review, with reasons for exclusions at each stage, ideally with a flow diagram. | Line 149 – 157: “To identify all published research assessing the role of genetic variation with dengue or WNV disease, we executed the above search strategy and identified 633 published reports (Table 1, Figure 1). Two researchers independently reviewed the titles and abstracts of these 633 papers and identified 104 papers for further full-text review in this meta-analysis (Table S2). One additional paper from 1987 was identified as pertinent during the ancestry search and was added to the review. The final analysis includes data from 88 of the 105 publications, following exclusion of 17 papers for cause (seven repeats, six conference abstracts, and four with an outcome other than disease severity).” |
| Study characteristics | 18 | For each study, present characteristics for which data were extracted (e.g., study size, PICOS, follow-up period) and provide the citations. | Supplemental Table 2 – Included Papers |
| Risk of bias within studies | 19 | Present data on risk of bias of each study and, if available, any outcome level assessment (see item 12). | Supplemental Table 5 – Quality Scores  Lines 239 – 244: “**Quality Scores**. Based on the Newcastle Ottawa Scoring System, the average quality score was 5.76 (range: 3 – 7) for the WNV publications and 5.10 (range: 2 – 7) for the dengue publications (Table S5). We also assessed whether the study authors corrected for multiple testing, and found less than half of both WNV and dengue studies provided corrected p-values when appropriate, indicating an inflated type I error rate.” |
| Results of individual studies | 20 | For all outcomes considered (benefits or harms), present, for each study: (a) simple summary data for each intervention group (b) effect estimates and confidence intervals, ideally with a forest plot. | Figures 2 and 3  Tables 2 and 3  Supplemental Tables 2, 3, 4 |
| Synthesis of results | 21 | Present results of each meta-analysis done, including confidence intervals and measures of consistency. | Figures 2 and 3 |
| Risk of bias across studies | 22 | Present results of any assessment of risk of bias across studies (see Item 15). | Line 319 – 322: “The number and type of genes examined varied greatly between studies, and we were limited by what genes researchers chose to sequence and include in publications.” |
| Additional analysis | 23 | Give results of additional analyses, if done (e.g., sensitivity or subgroup analyses, meta-regression [see Item 16]). | N/A |
| **DISCUSSION** | | |  |
| Summary of evidence | 24 | Summarize the main findings including the strength of evidence for each main outcome; consider their relevance to key groups (e.g., healthcare providers, users, and policy makers). | Lines 248 – 256: “Among the 88 studies examined, a wide range of genetic targets was found to be significant, with many of the genes unsurprisingly playing a key role in the immune system defense against viral infections.  Despite the large number of studies, only 21 genes were studied by more than one research group for an association with either disease. Throughout these studies, several key genes rose to the forefront as the most studied and the most significant associations. Many studies focused on the HLA region of the genome, and, although inconsistencies in data presentation preclude a meta-analyze of these results, there were clear signs of the importance of this area for both diseases.”  Lines 302 – 305: “When we meta-analyzed the dengue studies, we found significant associations between dengue disease and genetic variation in MBL2, PLCE1, IFN**-**γ, and MICB. The role of many of these genes in disease pathogenesis has been characterized through *in vivo* and *in vitro* studies.” |
| Limitations | 25 | Discuss limitations at study and outcome level (e.g., risk of bias), and at review-level (e.g., incomplete retrieval of identified research, reporting bias). | Lines 311 – 323: “Our study is limited by several factors, most notably by the available literature. To ensure we found as many papers as possible, we constructed a search strategy that involved multiple databases, used both subject headings and text words, was not limited to English articles, and included an ancestry search [102]. Despite our focus on significant results and genes studied by at least two research groups, the wide heterogeneity among the populations studied limited our ability to interpret the meta-analyzed results. The diversity of results among studies that examined the same SNP could be due to population heterogeneity, as well as to differences in study approach, including selection of control and comparison groups. The number and type of genes examined varied greatly between studies, and we were limited by what genes researchers chose to sequence and include in publications. The unavailability of comparable genotype data and the incomparability of research groups across some studies preclude a more in depth analysis at present.” |
| Conclusions | 26 | Provide a general interpretation of the results in the context of other evidence, and implications for future research. | Lines 324 – 336: “The genes found to be significantly associated with WNV or dengue disease pathogenesis varied in function, with most being linked to the immune response. As the regions of the world affected by WNV, dengue, and related viruses such as zika, continue to expand due in part to climate change, an improved understanding of the association between genetic variation and disease severity will be valuable for all potentially affected populations [103-106]. Based on the growing incidence of these diseases, the paucity of consistency in the associations found, and the limited overlap in genetic targets studied to date, there is need for continued and deeper studies examining the role of genetic factors in WNV and dengue disease severity. In addition to conducting new studies such as whole-exome sequencing within larger population samples, further analyses could be conducted of existing data, to glean novel findings such as gene-gene or gene-environment interactions, rare and low frequency variants, and pathways of significant determinants of anti-viral resistance.” |
| **FUNDING** | | |  |
| Funding | 27 | Describe sources of funding for the systematic review and other support (e.g., supply of data); role of funders for the systematic review. | Posted in application as requested |

*From:*  Moher D, Liberati A, Tetzlaff J, Altman DG, The PRISMA Group (2009). Preferred Reporting Items for Systematic Reviews and Meta-Analyses: The PRISMA Statement. PLoS Med 6(7): e1000097. doi:10.1371/journal.pmed1000097

For more information, visit: **www.prisma-statement.org**.

Page 2 of 2
