# Supplementary material for: Identification of genetic variants associated with dengue or West Nile virus disease: a systematic review and meta-analysis
Source: BMC Infect Dis. 2018 Jun 22;18:282. doi: 10.1186/s12879-018-3186-6 (PMC6014009; doi:10.1186/s12879-018-3186-6)
Supplement: Supplementary file 2 — Sample Search Strategy for the Embase Database. Medline, PubMed, Embase, and Global Health databases were used to search the literature. Search terms included West Nile or Dengue and genetic factors; the same set of text words was used for all databases in conjunction with subject headings that were tailored for each database. As an example, this search strategy for the Embase database is provided. These subject headings, in conjunction with the text search (Table 1), were used to find relevant literature in Embase. (DOCX 13 kb) [file 12879_2018_3186_MOESM2_ESM.docx]

1. exp single nucleotide polymorphism/
2. exp host susceptibility/
3. exp genetic polymorphism/
4. exp genetic risk/
5. exp genetic association/ or exp genome-wide association study/
6. exp genetic variability/
7. exp microsatellite marker/
8. exp dengue/ or exp Dengue virus 1/ or exp Dengue virus 2/ or exp Dengue virus 3/ or exp Dengue virus 4
9. exp West Nile virus/ or exp West Nile flavivirus/ or exp West Nile fever/
10. 8 or 9
11. 1 or 2 or 3 or 4 or 5 or 6 or 7
12. 10 and 11
