# Supplementary material for: Identification of genetic variants associated with dengue or West Nile virus disease: a systematic review and meta-analysis
Source: BMC Infect Dis. 2018 Jun 22;18:282. doi: 10.1186/s12879-018-3186-6 (PMC6014009; doi:10.1186/s12879-018-3186-6)
Supplement: Supplementary file 4 — HLA Associations with DENV Disease Severity. HLA alleles studied by two or more research groups for association with DENV disease severity. (DOCX 311 kb) [file 12879_2018_3186_MOESM4_ESM.docx]

| **Allele** | | **Cases** | **Comparison Group** | **Country** | **Key Results** |
| --- | --- | --- | --- | --- | --- |
| A | A*01 | 42 DHF | 67 DF | Brazil | OR of 2.75 (1.2 – 6.2)[1] |
|  |  | 110 DHF | 119 Controls | Sri Lanka | OR = 1.35 (0.76 – 2.41)[2] |
|  |  | 29 DHF & 85 DF | 110 Controls | India | 20.69% among DHF, 25.88% among DF, and 20% among controls. Between DF & DHF cases and controls, calculated allelic OR of 1.30[3] |
|  |  | 93 Symptomatic infections | 173 Controls | Brazil | 6.3% among cases and 11% among controls, for calculated allelic OR of 0.55[4] |
|  |  | 120 DF & DHF cases | 120 Controls | Cuba | 5.5% among cases and 6.7% among controls, for calculated allelic OR of 0.81[5] |
|  |  | 92 DF & DHF cases | 95 Controls | Malaysia | 5.43% among cases and 6.84% among controls, for calculated allelic OR of 0.78 [6] |
|  |  | 87 DHF | 138 Controls | Thailand | Significant association (p<0.05) with disease[7] |
|  |  | 82 DHF/DSS | 276 Controls | Cuba | Significant association (p<0.001) with disease[8] |
|  |  | 89 severe, 39 neuroinvasive | 63 asymptomatic | US & Canada | 38.1% among asymptomatic, 33.7% among severe, 40.0% among neuroinvasive[9] |
|  | A*02 | 42 DHF | 67 DF | Brazil | 0.73 (0.3 – 1.5) times odds of developing DHF over DF[1] |
|  |  | 309 DHF | 251 Controls | Vietnam | 42.4% cases and 49.4% controls, for calculated allelic OR of 0.75[10] |
|  |  | 110 DHF | 119 Controls | Sri Lanka | OR = 0.84 (0.50 – 1.40)[2] |
|  |  | 42 DHF | 67 DF | Brazil | OR = 0.73 (0.3 – 1.5)[1] |
|  |  | 29 DHF & 85 DF | 110 Controls | India | 41.38% among DHF, 23.53% among DF, and 35.45% among controls. Between DHF and DF, OR of 2.29 (0.84–6.09)[3] |
|  |  | 93 Symptomatic infections | 173 Controls | Brazil | 23.2% among cases and 28.3% among controls, for calculated allelic OR of 0.95[4] |
|  |  | 120 DF & DHF | 120 Controls | Cuba | 22.7% among cases and 18.1% among controls, for calculated allelic OR of 1.33[5] |
|  |  | 92 DF & DHF cases | 95 Controls | Malaysia | 20.65% among cases and 20% among controls, for calculated allelic OR of 1.04[6] |
|  |  | 87 DHF | 138 Controls | Thailand | Significant association (p<0.05) with disease[7] |
|  |  | 89 severe, 39 neuroinvasive | 63 asymptomatic | US & Canada | 49.2% among asymptomatic, 48.3% among severe, 40.0% among neuroinvasive[9] |
|  | A*0203 | 149 DF and 114 DHF | 140 Controls | Thailand | 17.5% among secondary DHF (n=103), 34.0% among secondary DF (n=106), 14.3% among controls. Between all secondary DF and controls, OR of 3.09 (1.59 - 6.02). Between secondary DHF and secondary DF, OR of 0.41 (0.20 - 0.82)[11] |
|  |  | 122 DSS, 34 DHF, 94 DF | 300 Controls | Philippines | 1.3% among DSS and DHF, 5.4% among DF, and 1% among controls. Between DSS/DHF and controls, OR of 1.3 (0.2–7.8). Between DF and controls, OR of 5.6 (1.3–24). Between DSS/DHF and DF, OR of 0.2 (0.04–1.2)[12] |
|  |  | 29 DHF & 85 DF | 110 Controls | India | 0% among DHF and DF, and 3.64% among controls[3] |
|  | A*03 | 92 DF & DHF cases | 95 Controls | Malaysia | 3.26% among cases and 9.47% among controls, for calculated allelic OR of 0.32[6] |
|  |  | 110 DHF | 119 Controls | Sri Lanka | OR of 1.32 (0.54 – 3.30)[2] |
|  |  | 42 DHF | 67 DF | Brazil | OR of 0.57 (0.1 – 1.8)[1] |
|  |  | 29 DHF & 85 DF | 110 Controls | India | 6.9% among DHF, 9.41% among DF, and 12.73% among controls. Between cases and controls, calculated allelic OR of 0.66[3] |
|  |  | 93 Symptomatic infections | 173 Controls | Brazil | 14.2% among cases and 8.7% among controls, for calculated allelic OR of 1.69[4] |
|  |  | 120 DF & DHF | 120 Controls | Cuba | 7% among cases and 8% among controls, for calculated allelic OR of 0.87[5] |
|  |  | 89 severe, 39 neuroinvasive | 63 asymptomatic | US & Canada | 28.6% among asymptomatic, 22.5% among severe, 22.5% among neuroinvasive[9] |
|  | A*11 | 42 DHF | 67 DF | Brazil | OR of 0.89 (0.20 – 2.7)[1] |
|  |  | 110 DHF | 119 Controls | Sri Lanka | OR of 1.04 (0.58 – 1.89)[2] |
|  |  | 29 DHF & 85 DF | 110 Controls | India | 41.38% among DHF, 23.53% among DF, and 35.45% among controls. Between DHF and DF, OR of 2.29 (0.84–6.09).[3] |
|  |  | 93 Symptomatic infections | 173 Controls | Brazil | 5.3% among cases and 4.6% among controls, for calculated allelic OR of 1.16[4] |
|  |  | 92 DF & DHF cases | 95 Controls | Malaysia | 25.54% among cases and 24.21% among controls, for calculated allelic OR of 1.07 [6] |
|  |  | 309 DHF | 251 Controls | Vietnam | 48.9% cases and 45.8% controls, for calculated allelic OR of 1.13[10] |
|  |  | 120 DF & DHF | 120 Controls | Cuba | 1% among cases and 2.7% among controls, for calculated allelic OR of 0.36[5] |
|  |  | 89 severe, 39 neuroinvasive | 63 asymptomatic | US & Canada | 12.7% among asymptomatic, 13.5% among severe, 20.0% among neuroinvasive[9] |
|  | A*24 | 309 DHF | 251 Controls | Vietnam | 35% among cases and 25.9% among controls, for calculated allelic OR of 1.54[10] |
|  |  | 110 DHF | 119 Controls | Sri Lanka | 24.55% among cases and 19.75% among controls, for OR of 1.32 (0.85 – 2.06)[2] |
|  |  | 42 DHF | 67 DF | Brazil | OR of 1.1 (0.6 – 2.2)[1] |
|  |  | 29 DHF & 85 DF | 110 Controls | India | 31.03% among DHF, 27.06% among DF, and 28.18% among controls. Between cases and controls, calculated allelic OR of 0.99[3] |
|  |  | 93 Symptomatic infections | 173 Controls | Brazil | 8.9% among cases and 9.5% among controls, for calculated allelic OR of 0.93[4] |
|  |  | 120 DF & DHF | 120 Controls | Cuba | 9.5% among cases and 9.2% among controls, for calculated allelic OR of 1.04[5] |
|  |  | 92 DF & DHF cases | 95 Controls | Malaysia | 25.54% among cases and 21.58% among controls, for calculated allelic OR of 1.26[6] |
|  |  | 89 severe, 39 neuroinvasive | 63 asymptomatic | US & Canada | 6.4% among asymptomatic, 13.5% among severe, 7.5% among neuroinvasive[9] |
|  | A*26 | 42 DHF | 67 DF | Brazil | OR of 0.30 (0.10 – 3.3)[1] |
|  |  | 110 DHF | 119 Controls | Sri Lanka | OR of 0.82 (0.35 – 1.92)[2] |
|  |  | 29 DHF & 85 DF | 110 Controls | India | 13.79% among DHF, 14.12% among DF, and 10.09% among controls. Between cases and controls, calculated allelic OR of 1.46.[3] |
|  |  | 93 Symptomatic infections | 173 Controls | Brazil | 3.7% among cases and 3.5% among controls, for calculated allelic OR of 1.06[4] |
|  |  | 92 DF & DHF cases | 95 Controls | Malaysia | 1.63% among cases and 1.05% among controls, for calculated allelic OR of 1.56[6] |
|  |  | 120 DF & DHF | 120 Controls | Cuba | 1.5% among cases and 3.1% among controls, for calculated allelic OR of 0.48[5] |
|  | A*31 | 120 DF & DHF | 120 Controls | Cuba | 4.5% among DF & DHF cases and 0.8% among controls, for OR of 7.60 (2.30 – 27.70). Between DHF cases and controls, OR of 13.39 (3.63–53.75)[5] |
|  |  | 110 DHF | 119 Controls | Sri Lanka | OR of 0.76 (0.28 – 2.00)[2] |
|  |  | 42 DHF | 67 DF | Brazil | OR of 0.5 (0.2 – 1.2)[1] |
|  |  | 29 DHF & 85 DF | 110 Controls | India | 0% among DHF, 4.71% among DF, 6.36% among controls. Between cases and controls, calculated allelic OR of 1.87[3] |
|  |  | 93 Symptomatic infections | 173 Controls | Brazil | 2.6% among cases and 3.8% among controls, for calculated allelic OR of 0.68[4] |
|  |  | 92 DF & DHF cases | 95 Controls | Malaysia | 2.17% among cases and 1.58% among controls, for calculated allelic OR of 1.38[6] |
|  |  | 89 severe, 40 neuroinvasive | 63 asymptomatic | US & Canada | 7.9% among asymptomatic, 3.4% among severe, 5.0% among neuroinvasive[9] |
|  | A*33 | 309 DHF | 251 Controls | Vietnam | 11% among cases and 18.3% among controls, for calculated allelic OR of 0.55[10] |
|  |  | 110 DHF | 119 Controls | Sri Lanka | OR of 0.96 (0.60 – 1.53)[2] |
|  |  | 42 DHF | 67 DF | Brazil | No elevated odds of developing disease (OR of 1.0 (0.3 – 9.4)[1] |
|  |  | 29 DHF & 85 DF | 110 Controls | India | 17.24% among DHF, 27.06% among DF, 17.27% among controls. Between cases and controls, calculated allelic OR of 1.56[3] |
|  |  | 93 Symptomatic infections | 173 Controls | Brazil | 2.6% among cases and controls (allelic OR of 1.00) [4] |
|  |  | 120 DF & DHF | 120 Controls | Cuba | 3.0% among cases and 5.4% among controls, for calculated allelic OR of 0.54[5] |
|  |  | 92 DF & DHF cases | 95 Controls | Malaysia | 7.61% among cases and 10.53% among controls, for calculated allelic OR of 0.70[6] |
|  |  | 122 DSS, 34 DHF, 94 DF | 300 Controls | Philippines | 2% among DSS and DHF, 5.4% among DF, and 11.5% among controls. Between DSS/DHF and controls, OR of 0.2 (0.05–0.5). Between DF and controls, OR of 0.4 (0.2–1.2). Between DSS/DHF and DF, OR of 0.3 (0.08–1.5)[12] |
| B | B*07 | DHF | Controls |  | OR of 1.60 (DHF vs controls) and 1.44 (secondary DHF + DF cases compared to controls)[13] |
|  |  | 110 DHF | 119 Controls | Sri Lanka | OR of 1.08 (0.55 – 2.15)[2] |
|  |  | 29 DHF & 85 DF | 110 Controls | India | 17.86% among DHF, 22.89% among DF, 24.55% among controls. Between cases and controls, calculated allelic OR of 0.85[3] |
|  |  | 93 Symptomatic infections | 173 Controls | Brazil | 4.7% among cases and 6.9% among controls, for calculated allelic OR of 0.67[4] |
|  |  | 120 DF & DHF | 120 Controls | Cuba | 7.0% among cases and 8.9% among controls for calculated allelic OR of 0.77[5] |
|  |  | 92 DF & DHF cases | 95 Controls | Malaysia | 5.98% among cases and 5.26% among controls, for calculated allelic OR of 1.15[6] |
|  |  | 122 DSS, 34 DHF, 94 DF | 300 Controls | Philippines | 1.9% among DSS and DHF, 4.3% among DF, and 6.1% among controls. Between DSS/DHF and controls, OR of 0.3 (0.09–1.1). Between DF and controls, OR of 0.7 (0.2–2.1). Between DSS/DHF and DF, 0.4 (0.1–2.0)[12] |
|  |  | 16 DHF & 23 DF | 34 Controls | Mexico | 4.3% among DF, 3.1% among DHF, and 1.4% among controls. [14] |
|  |  | 89 severe, 39 neuroinvasive | 63 asymptomatic | US & Canada | 22.2% among asymptomatic, 24.7% among severe, 23.1% among neuroinvasive[9] |
|  | B*13 | 110 DHF | 119 Controls | Sri Lanka | OR = 1.99 (0.66 – 6.03)[2] |
|  |  | 29 DHF & 85 DF | 110 Controls | India | 0% among DHF, 6.02% among DF, and 8.18% among controls. Between cases and controls, calculated allelic OR of 1.69[3] |
|  |  | 93 Symptomatic infections | 173 Controls | Brazil | 2.6% among cases and 2.0% among controls, for calculated allelic OR of 1.31[4] |
|  |  | 120 DF & DHF | 120 Controls | Cuba | 1.5% among cases and controls[5] |
|  |  | 92 DF & DHF cases | 95 Controls | Malaysia | 9.78% among cases and 6.32% among controls, for calculated allelic OR of 1.61[6] |
|  |  | 87 DHF | 138 Controls | Thailand | Significant association (p<0.05) with disease[7] |
|  |  | 89 severe, 39 neuroinvasive | 63 asymptomatic | US & Canada | 7.9% among asymptomatic, 5.6% among severe, 2.6% among neuroinvasive[9] |
|  | B*15 | 120 DF & DHF | 120 Controls | Cuba | 15.6% among cases and 4.4% among controls. Between cases and controls, OR of 3.36 (1.96 – 10.29); between DF and controls, OR of 4.77 (1.88-12.21); between DHF and controls, 4.07 (1.46 – 11.36)[5] |
|  |  | 110 DHF | 119 Controls | Sri Lanka | OR of 0.98 (0.51 – 1.87)[2] |
|  |  | 29 DHF & 85 DF | 110 Controls | India | 7.14% among DHF, 18.07% among DF, 13.64% among controls. Between cases and controls, calculated allelic OR of 1.15[3] |
|  |  | 93 Symptomatic infections | 173 Controls | Brazil | 9.5% among cases and 8.1% among controls, for calculated allelic OR of 1.19[4] |
|  |  | 92 DF & DHF cases | 95 Controls | Malaysia | 14.67% among cases and 12.63% among controls, for calculated allelic OR of 1.19[6] |
|  |  | 16 DHF & 23 DF | 34 Controls | Mexico | 8.6% among DF, 6.2% among DHF, and 11.7% among controls[14] |
|  |  | 89 severe, 39 neuroinvasive | 63 asymptomatic | US & Canada | 9.5% among asymptomatic, 14.6% among severe, 7.7% among neuroinvasive[9] |
|  | B*35 | 16 DHF & 23 DF | 34 Controls | Mexico | 10.8% among DF, 9.3% among DHF, and 33.8% among controls. Between dengue cases and controls, OR of 0.12 (0.037 – 0.39). Between DF and controls, OR of 0.13 (0.031 – 0.51)[14] |
|  |  | 29 DHF & 85 DF | 110 Controls | India | 25% among DHF, 25.30% among DF, 16.36% among controls. Between cases and controls, calculated allelic OR of 1.81[3] |
|  |  | 93 Symptomatic infections | 173 Controls | Brazil | 12.1% among cases and 14.2% among controls, for calculated allelic OR of 0.83[4] |
|  |  | 120 DF & DHF | 120 Controls | Cuba | 8.0% among cases and 9.8% among controls, for calculated allelic OR of 0.80[5] |
|  |  | 92 DF & DHF cases | 95 Controls | Malaysia | 7.61% among cases and 12.11% among controls, for calculated allelic OR of 0.60[6] |
|  |  | 122 DSS, 34 DHF, 94 DF | 300 Controls | Philippines | 14.8% among DSS/DHF, 7.5% among DF, 8.1% among controls. Between DSS/DHF and controls, 2.0 (1.1–3.6). Between DF and controls, OR of 0.9 (0.4–2.2). Between DSS/DHF and DF, OR of 2.1 (0.9–5.2)[12] |
|  |  | 110 DHF | 119 Controls | Sri Lanka | OR of 1.49 (0.86 – 2.58)[2] |
|  |  | 89 severe, 39 neuroinvasive | 63 asymptomatic | US & Canada | 20.6% among asymptomatic, 14.6% among severe, 23.1% among neuroinvasive[9] |
|  | B*44 | 16 DHF & 23 DF | 34 Controls | Mexico | 2.1% among DF, 3.1% among DHF, and 4.4% among controls[14] |
|  |  | 110 DHF | 119 Controls | Sri Lanka | OR of 1.09 (0.61 – 1.95)[2] |
|  |  | 29 DHF & 85 DF | 110 Controls | India | 14.28% among DHF, 19.28% among DF, and 18.18% among controls. Between cases and controls, calculated allelic OR of 1.05[3] |
|  |  | 93 Symptomatic infections | 173 Controls | Brazil | 10.5% among cases and 7.8% among controls, for calculated allelic OR of 1.39[4] |
|  |  | 120 DF & DHF | 120 Controls | Cuba | 8.5% among cases and 9.7% among controls, for calculated allelic OR of 0.86[5] |
|  |  | 92 DF & DHF cases | 95 Controls | Malaysia | 1.63% among cases and 2.63% among controls, for calculated allelic OR of 0.61[6] |
|  |  | 149 DF and 114 DHF | 140 Controls | Thailand | 9.3% primary DF (n=43), 11.3% secondary DF (n=106), 18.2% primary DHF (n=11), 4.9% secondary DHF (n=103), 15% controls. Between DHF with secondary infections and controls, OR of 0.29 (0.09 - 0.85)[11] |
|  |  | 89 severe, 39 neuroinvasive | 63 asymptomatic | US & Canada | 19.1% among asymptomatic, 23.6% among severe, 33.3% among neuroinvasive[9] |
|  |  | 89 severe, 39 neuroinvasive | 63 asymptomatic | US & Canada | 19.1% among asymptomatic, 23.6% among severe, 33.3% among neuroinvasive[9] |
|  | B*48 | 93 Symptomatic infections | 173 Controls | Brazil | 1.6% among cases and 0% among controls[4] |
|  |  | 120 DF & DHF | 120 Controls | Cuba | 0.5% among cases and 0% among controls[5] |
|  |  | 92 DF & DHF cases | 95 Controls | Malaysia | 2.72% among cases and 0.53% among controls for calculated allelic OR of 5.25[6] |
|  |  | 16 DHF & 23 DF | 34 Controls | Mexico | 4.3% among DF, 9.3% among DHF, and 4.4% among controls[14] |
|  |  | 122 DSS, 34 DHF, 94 DF | 300 Controls | Philippines | 7.1% among DSS/DHF, 2.2% among DF, 8.1% among controls. Between DSS/DHF and controls, OR of 0.9 (0.4–1.8). Between DF and controls, OR of 0.3 (0.06–1.1). Between DSS/DHF and DF, OR of 3.5 (0.8–16.0)[12] |
|  | B*51 | 149 DF and 114 DHF | 140 Controls | Thailand | 5.7% of secondary DF (n=106), 15.5% secondary DHF (n=103) and 4.3% controls. Between DHF and DF, OR of 3.07 (1.07- 9.22). Between DHF and controls, OR of 4.11 (1.44-12.28)[11] |
|  |  | 16 DHF & 23 DF | 34 Controls | Mexico | 0% among DF, 3.1% among DHF, and 5.8% among controls[14] |
|  |  | 110 DHF | 119 Controls | Sri Lanka | OR of 0.95 (0.46 – 1.95)[2] |
|  |  | 29 DHF & 85 DF | 110 Controls | India | 10.71% among DHF, 15.7% among DF, and 11.82% among controls. Between cases and controls, calculated allelic OR of 1.26[3] |
|  |  | 93 Symptomatic infections | 173 Controls | Brazil | 8.4% among cases and 11.8% among controls, for calculated allelic OR of 0.69[4] |
|  |  | 120 DF & DHF | 120 Controls | Cuba | 3% among cases and 4.7% among controls, for calculated allelic OR of 1.59[5] |
|  |  | 92 DF & DHF cases | 95 Controls | Malaysia | 6.52% among cases and 5.79% among controls, for calculated allelic OR of 1.13[6] |
|  |  | 122 DSS, 34 DHF, 94 DF | 300 Controls | Philippines | 10.3% among DSS/DHF, 6.5% among DF, 5.1% among controls. Between DSS/DHF and controls, OR of 2.2 (1.0–4.5). Between DF and controls, OR of 1.3 (0.5–3.4). Between DSS/DHF and DF, OR of 1.7 (0.6–4.4)[12] |
|  |  | 89 severe, 39 neuroinvasive | 63 asymptomatic | US & Canada | 11.1% among asymptomatic, 6.7% among severe, 5.1% among neuroinvasive[9] |
|  | B*52 | 149 DF and 114 DHF | 140 Controls | Thailand | 11.3% of secondary DF (n=106), 1% secondary DHF (n=103) and 2.1% controls. Between secondary DF and controls, OR of 5.83 (1.48 - 26.80). Between secondary DHF and controls, OR of 0.08 (0.00 - 0.59)[11] |
|  |  | 110 DHF | 119 Controls | Sri Lanka | OR of 1.18 (0.52 – 2.65)[2] |
|  |  | 29 DHF & 85 DF | 110 Controls | India | 10.71% among DHF, 12.05% among DF, and 15.45% among controls. Between cases and controls, calculated allelic OR of 0.73[3] |
|  |  | 93 Symptomatic infections | 173 Controls | Brazil | 3.2% among cases and 1.2% among controls, for calculated allelic OR of 2.72[4] |
|  |  | 120 DF & DHF | 120 Controls | Cuba | 0.5% among cases and 0.8% among controls, for calculated allelic OR of 0.62[5] |
|  |  | 92 DF & DHF cases | 95 Controls | Malaysia | 5.43% among cases and 4.21% among controls, for calculated allelic OR of 1.31[6] |
|  |  | 16 DHF & 23 DF | 34 Controls | Mexico | 6.5% among DF, 6.2% among DHF, and 1.4% among controls[14] |
|  | B*57 | 110 DHF | 119 Controls | Sri Lanka | OR of 0.93 (0.49 – 1.77)[2] |
|  |  | 29 DHF & 85 DF | 110 Controls | India | 10.71% among DHF, 6.02% among DF, and 8.18% among controls. Between cases and controls, calculated allelic OR of 0.87[3] |
|  |  | 93 Symptomatic infections | 173 Controls | Brazil | 3.2% among cases and 1.4% among controls, for calculated allelic OR of 2.33[4] |
|  |  | 120 DF & DHF | 120 Controls | Cuba | 4% among cases and 2.7% among controls, for calculated allelic OR of 1.50[5] |
|  |  | 16 DHF & 23 DF | 34 Controls | Mexico | 4.3% among DF, 0% among DHF, and 1.4% among controls[14] |
|  |  | 92 DF & DHF cases | 95 Controls | Malaysia | 3.8% among cases and 2.63% among controls, for calculated allelic OR of 1.46[6] |
|  |  | 89 severe, 39 neuroinvasive | 63 asymptomatic | US & Canada | 11.1% among asymptomatic, 4.5% among severe, 12.8% among neuroinvasive[9] |
| C | C*07 | 93 Symptomatic infections | 173 Controls | Brazil | 28% among cases and 22.7% among controls, for OR of 0.59 (0.37 – 0.95)[4] |
|  |  | 82 severe, 29 neuroinvasive | 66 asymptomatic | US & Canada | 59.1% among asymptomatic, 53.1% among severe, 55.2% among neuroinvasive[9] |
|  | Cw*07 | 110 DHF | 119 Controls | Sri Lanka | 24.37% among controls, 25.91% among cases, for OR of 1.09 (0.71 – 1.66)[2] |
|  |  | 29 DHF & 85 DF | 110 Controls | India | 62.06% among DHF, 56.09% among DF, and 40.56% among controls. Between cases and controls, calculated allelic OR of 1.99[3] |
|  |  | 120 DF & DHF | 120 Controls | Cuba | 15.1% among cases and 15.5% among controls, for calculated allelic OR of 0.97[5] |
| DRB1 | *04 | 47 DF & 34 DHF | 99 Controls | Mexico | 35.2% among DHF, 63.8% among DF, and 23.7% among controls. Between DHF and DF, OR of 0.31 (0.11–0.85)[15] |
|  |  | 85 DF & 29 DHF | 110 Controls | India | 10.3% among DHF, 20.0% among DF, and 15.4% among controls[16] |
|  |  | 93 Symptomatic infections | 173 Controls | Brazil | 13.2% among cases and 9.5% among controls, for calculated allelic OR of 1.45[4] |
|  |  | 110 DHF | 119 Controls | Sri Lanka | OR of 1.31 (0.66 – 2.62)[2] |
|  |  | 120 DF & DHF | 120 Controls | Cuba | 8.1% among cases and 14.2% among controls, for calculated allelic OR of 0.53[5] |
|  | *06:11 | 16 DHF & 23 DF | 34 Controls | Mexico | 0% among DHF, 6.5% among DF, and 1.4% among controls[14] |
|  |  | 120 DF & DHF | 120 Controls | Cuba | 7.0% among cases and 19.7% among controls. Between cases and controls, OR of 0.25 (0.11–0.55); between DHF and controls, 0.27 (0.09–0.77); between DF and controls, 0.24 (0.08–0.63)[5] |
|  |  | 93 Symptomatic infections | 173 Controls | Brazil | 12.1% among cases and 11% among controls, for calculated allelic OR of 1.11[4] |
|  |  | 110 DHF | 119 Controls | Sri Lanka | OR of 1.16 (0.75 – 1.81)[2] |
|  |  | 47 DF & 34 DHF | 99 Controls | Mexico | 11.7% among DHF, 12.7% among DF, and 11.1% among controls[15] |
|  |  | 85 DF & 29 DHF | 110 Controls | India | 44.8% among DHF, 17.6% among DF, and 21.8% among controls[16] |
|  | *07 | 10 primary DHF, 59 primary DF, 132 secondary DH 169 secondary DF | 65 asymptomatic | Thailand | Identified in some patients[17] |
|  |  | 120 DF & DHF | 120 Controls | Cuba | 7.0% among cases and 19.7% among controls[5] |
|  |  | 122 DSS, 34 DHF, 94 DF | 300 Controls | Philippines | 6.7% among DSS, 6.1% among DHF, 7.8% among DF, 9.3% among controls[12] |
|  |  | 93 Symptomatic infections | 173 Controls | Brazil | 12.1% among cases and 11.0% among controls[4] |
|  | *09 | 122 DSS, 34 DHF, 94 DF | 300 Controls | Philippines | 11.68% among DSS, 12.1% among DHF, 10.0% among DF, 16.0% among controls[12] |
|  |  | 93 Symptomatic infections | 173 Controls | Brazil | 3.7% among cases and 1.2% among controls, for calculated allelic OR of 3.16[4] |
|  |  | 120 DF & DHF | 120 Controls | Cuba | 4.3% among cases and 3.2% among controls, for calculated allelic OR of 1.36[5] |
|  |  | 47 DF & 34 DHF | 99 Controls | Mexico | 5.8% among DHF, 0% among DF, 1.5% among controls[15] |
|  |  | 85 DF & 29 DHF | 110 Controls | India | 0% among DHF, 4.7% among DF, and 0.91% among controls[16] |
|  | *10 | 110 DHF | 119 Controls | Sri Lanka | OR = 0.96 (0.48 – 1.93)[2] |
|  |  | 29 DHF & 85 DF | 110 Controls | India | 10.3% among DHF, 11.7% among DF, 20% among controls[3] |
|  |  | 93 Symptomatic infections | 173 Controls | Brazil | 2.1% among cases and 2.3% among controls, for calculated allelic OR of 0.91[4] |
|  |  | 120 DF & DHF | 120 Controls | Cuba | 3.8% among cases and 1.2% among controls, for calculated allelic OR of 3.25[5] |
|  |  | DF & DHF | Controls | Mexico | 2.9% among DHF, 2.1% among DF, 0.5% among controls[15] |
|  |  | 85 DF & 29 DHF | 110 Controls | India | 10.3% among DHF, 11.7% among DF, and 20.0% among controls[16] |
|  | *11 | 93 Symptomatic infections | 173 Controls | Brazil | 8.4% among cases and 16.2% among controls, for an OR of 0.48 (0.27 – 0.86)[4] |
|  |  | 120 DF & DHF | 120 Controls | Cuba | 4.8% among cases and 8.8% among controls, for calculated allelic OR of 0.52[5] |
|  |  | 47 DF & 34 DHF | 99 Controls | Mexico | 11.7% among DHF, 2.1% among DF, 10.1% among controls. Between DF and controls, OR of 0.09, (0.0–0.64)[15] |
|  |  | 85 DF & 29 DHF | 110 Controls | India | 13.8% among DHF, 4.7% among DF, and 7.3% among controls[16] |
|  |  | 110 DHF | 119 Controls | Sri Lanka | OR = 1.99 (0.66 – 6.03)[2] |
|  | *15 | 93 Symptomatic infections | 173 Controls | Brazil | 11.1% among cases and 5.5% among controls, for OR of 2.14 (1.12 – 4.09)[4] |
|  |  | 29 DHF & 85 DF | 110 Controls | India | 51.8% among DF, 51.72% among DHF, 49.1% among controls[3] |
|  |  | 110 DHF | 119 Controls | Sri Lanka | OR = 0.99 (0.64 – 1.56)[2] |
|  |  | 85 DF & 29 DHF | 110 Controls | India | 51.72% among DHF, 51.8% among DF, and 49.1% among controls[16] |
|  |  | 120 DF & DHF | 120 Controls | Cuba | 5.4% among cases and 6.4% among controls, for calculated allelic OR of 0.83[5] |

References

1. Monteiro SP, Brasil PE, Cabello GM, Souza RV, Brasil P, Georg I, et al. HLA-A*01 allele: a risk factor for dengue haemorrhagic fever in Brazil's population. Memorias do Instituto Oswaldo Cruz. 2012;107(2):224-30. PubMed PMID: 366395351.

2. Malavige GN, Rostron T, Rohanachandra LT, Jayaratne SD, Fernando N, Silva ADd, et al. HLA class I and class II associations in dengue viral infections in a Sri Lankan population. PLoS ONE. 2011. PubMed PMID: 20113251877.

3. Alagarasu K, Mulay AP, Sarikhani M, Rashmika D, Shah PS, Cecilia D. Profile of human leukocyte antigen class I alleles in patients with dengue infection from Western India. Human Immunology. 2013;74(12):1624-8. PubMed PMID: 23974055.

4. Cardozo DM, Moliterno RA, Sell AM, Guelsin GA, Beltrame LM, Clementino SL, et al. Evidence of HLA-DQB1 Contribution to Susceptibility of Dengue Serotype 3 in Dengue Patients in Southern Brazil. Journal of Tropical Medicine. 2014;2014:968262. PubMed PMID: 24817893.

5. Sierra B, Alegre R, Perez AB, Garcia G, Sturn-Ramirez K, Obasanjo O, et al. HLA-A, -B, -C, and -DRB1 allele frequencies in Cuban individuals with antecedents of dengue 2 disease: advantages of the Cuban population for HLA studies of dengue virus infection. Human Immunology. 2007;68(6):531-40. PubMed PMID: 17509453.

6. Appanna R, Ponnampalavanar S, Lum Chai See L, Sekaran SD. Susceptible and protective HLA class 1 alleles against dengue fever and dengue hemorrhagic fever patients in a Malaysian population.[Erratum appears in PLoS One. 2011;6(1). doi: 10.1371/annotation/972cc8f0-5c9e-4d74-b98b-217a2f80a4bd]. PLoS ONE [Electronic Resource]. 2010;5(9):28. PubMed PMID: 20927388.

7. Chiewsilp P, Scott RM, Bhamarapravati N. Histocompatibility antigens and dengue hemorrhagic fever. Am J Trop Med Hyg. 1981;30(5):1100-5. Epub 1981/09/01. PubMed PMID: 7283007.

8. Paradoa Perez ML, Trujillo Y, Basanta P. Association of dengue hemorrhagic fever with the HLA system. Haematologia (Budap). 1987;20(2):83-7. PubMed PMID: 3498669.

9. Lanteri MC, Kaidarova Z, Peterson T, Cate S, Custer B, Wu S, et al. Association between HLA class i and class ii alleles and the outcome of West Nile Virus infection: An exploratory study. PLoS ONE. 2011;6 (8) (no pagination)(e22948). PubMed PMID: 362247180.

10. Loke H, Bethell DB, Phuong CX, Dung M, Schneider J, White NJ, et al. Strong HLA class I--restricted T cell responses in dengue hemorrhagic fever: a double-edged sword? Journal of Infectious Diseases. 2001;184(11):1369-73. PubMed PMID: 11709777.

11. Stephens HAF, Klaythong R, Sirikong M, Vaughn DW, Green S, Kalayanarooj S, et al. HLA-A and -B allele associations with secondary dengue virus infections correlate with disease severity and the infecting viral serotype in ethnic Thais. Tissue Antigens. 2002;60(4):309-18. PubMed PMID: 36020803.

12. Mercado ES, Espino FE, Perez Ma LM, Bilar JM, Bajaro JDP, Huy NT, et al. HLA-A*33:01 as protective allele for severe dengue in a population of Filipino children. PLoS ONE. 2015;10 (2) (no pagination)(e0115619). PubMed PMID: 602151125.

13. Vejbaesya S, Thongpradit R, Kalayanarooj S, Luangtrakool K, Luangtrakool P, Gibbons RV, et al. HLA Class I Supertype Associations With Clinical Outcome of Secondary Dengue Virus Infections in Ethnic Thais. Journal of Infectious Diseases. 2015;212(6):939-47. PubMed PMID: 25740956.

14. Falcon-Lezama JA, Ramos C, Zuniga J, Juarez-Palma L, Rangel-Flores H, Garcia-Trejo AR, et al. HLA class I and II polymorphisms in Mexican Mestizo patients with dengue fever. Acta Tropica. 2009;112(2):193-7. PubMed PMID: 19653987.

15. LaFleur C, Granados J, Vargas-Alarcon G, Ruiz-Morales J, Villarreal-Garza C, Higuera L, et al. HLA-DR antigen frequencies in Mexican patients with dengue virus infection: HLA-DR4 as a possible genetic resistance factor for dengue hemorrhagic fever. Human Immunology. 2002;63(11):1039-44. PubMed PMID: 12392857.

16. Alagarasu K, Mulay AP, Singh R, Gavade VB, Shah PS, Cecilia D. Association of HLA-DRB1 and TNF genotypes with dengue hemorrhagic fever. Human Immunology. 2013;74(5):610-7. PubMed PMID: 52464744.

17. Vejbaesya S, Luangtrakool P, Luangtrakool K, Kalayanarooj S, Vaughn DW, Endy TP, et al. TNF and LTA gene, allele, and extended HLA haplotype associations with severe dengue virus infection in ethnic Thais. Journal of Infectious Diseases. 2009;199(10):1442-8. PubMed PMID: 20093154521.
